# There and back again: the shape of telemedicine in U.S. nursing homes following COVID-19

**DOI:** 10.1186/s12877-022-03046-y

**Published:** 2022-04-19

**Authors:** James H Ford, Sally A Jolles, Dee Heller, Madeline Langenstroer, Christopher Crnich

**Affiliations:** 1 Social & Administrative Sciences Division, School of Pharmacy, University of Wisconsin, 777 Highland Ave, Madison, WI 53705 USA; 2 University of Wisconsin School of Medicine & Public Health, Madison, WI USA; 3 William S. Middleton VA Hospital, Madison, WI USA

**Keywords:** Telemedicine, Organizational enhancements, Nursing homes, SEIPs model, Mixed methods, Providers, Nursing home staff

## Abstract

**Introduction:**

Telemedicine use in nursing homes (NHs) expanded during the COVID-19 pandemic. The objectives of this study were to characterize plans to continue telemedicine among newly adopting NHs and identify factors limiting its use after COVID-19.

**Methods:**

Key informants from 9 Wisconsin NHs that adopted telemedicine during COVID-19 were recruited. Semi-structured interviews and surveys were employed to identify participant perceptions about the value of telemedicine, implementation challenges encountered, and plans and barriers to sustaining its delivery after COVID-19. Directed content analysis and a deductive thematic approach using the Systems Engineering Initiative for Patient Safety (SEIPS) model was used during analyses. Quantitative and qualitative data were integrated to identify participant views on the value of telemedicine and the tools and work system enhancements needed to make telemedicine easier and more effective.

**Results:**

All participating NHs indicated a preference to continue telemedicine after COVID-19. Urgent assessments of resident change-in-condition and cognitively based sub-specialty consultations were identified as the encounter types most amenable to telemedicine. Reductions in resident off-site encounters and minimization of resident therapy interruptions were identified as major benefits of telemedicine. Twelve work system enhancements needed to better sustain telemedicine were identified, including improvements to: 1) equipment/IT infrastructure; 2) scheduling; 3) information exchange; and 4) telemedicine facilitators.

**Discussion:**

NHs that adopted telemedicine during COVID-19 wish to continue its use. However, interventions that enhance the integration of telemedicine into NH and off-site clinic work systems require changes to existing regulations and reimbursement models to sustain its utilization after COVID-19.

**Supplementary Information:**

The online version contains supplementary material available at 10.1186/s12877-022-03046-y.

## Introduction

Nursing homes (NHs) were the epicenter for the beginning of the coronavirus disease 2019 (COVID-19) pandemic in the United States [1, 2]. Nearly 33% of all documented COVID-19 related deaths in the United States occurred among residents of NHs, which represent only 0.4% of the U.S. population [3]. In an effort to reduce the spread of severe acute respiratory syndrome coronavirus 2 (SARS-CoV-2) in NHs, the Center for Medicare and Medicaid Services (CMS) implemented wide sweeping policies to curtail face-to-face clinical encounters and remove regulatory barriers to telemedicine [4].

Perhaps unsurprisingly, telemedicine activity expanded dramatically in U.S. NHs during the COVID-19 pandemic [5]. While relaxation of regulatory barriers undoubtedly played a role in this expansion [6], safety concerns have likely played an equally important role. Despite compelling evidence of beneficial effects on reducing potentially avoidable hospitalizations and expanding resident access to sub-specialty services [7, 8], telemedicine utilization was low in NHs prior to COVID-19 [9, 10]. Achieving a better understanding of staff and provider perceptions of the value of telemedicine and existing barriers to its delivery may help identify strategies to sustain its use in NHs that have newly adopted this technology. Without this knowledge, there is a risk of telemedicine de-adoption in NHs as safety concerns around COVID-19 abate.

Our research team recently embarked on a mixed-methods work system study in Wisconsin NHs that newly adopted or greatly expanded telemedicine services during the COVID-19 pandemic. The objectives of this study were to: 1) characterize facility plans for continuing telemedicine following COVID-19; 2) characterize staff and provider perspectives on the value and utility of telemedicine; and 3) identify the barriers NHs face with conducting telemedicine encounters. Based on the findings from this study, we discuss key work system changes that must occur to better sustain widespread utilization of telemedicine in NHs after COVID-19.

## Methodology

### Study design, setting, and participants

We conducted a mixed methods convergent study [11] of telemedicine use in NHs located in South Central Wisconsin. A convenience sample of nine NHs that had newly adopted or significantly expanded telemedicine during the COVID-19 pandemic were purposively selected for this study. Members of participating NH nursing administrative staff, long-term care advanced practice providers (APPs) that provided NH care in the region and sub-specialty care providers in the same region were purposively recruited. Participating NH staff were either the Director of Nursing, Associate Director of Nursing, Nursing Home Administrator, Unit Coordinator, Volunteer Services Coordinator, and/or Regional RN. Participating APPs and sub-specialists were employed by the same regional healthcare system, and all had conducted telemedicine encounters in at least one NH during the COVID-19 pandemic. This project was certified as a quality improvement by the UW-Madison Health Science Institutional Review Board. Informed consent was obtained for study participation from all participants.

### Data collection

Data collection from members of the NH administrative staff was conducted using semi-structured interviews. These Interviews focused on the following areas: 1) facility experience and challenges with implementing and using telemedicine during COVID-19; 2) facility plans for using telemedicine after COVID-19; 3) the relative advantages and disadvantages of telemedicine versus face-to-face encounters; 4) the types of resident encounters most amenable to telemedicine; and 5) the tools and resources that can make telemedicine encounters easier and/or more effective. Data collection from APP and sub-specialty provider participants was achieved through a structured survey and semi-structured interviews, respectively. A survey approach was employed with APP participants as prior interviews with these providers had provided our team with a detailed understanding of how they conducted telemedicine encounters. In contrast, understanding of how sub-specialty providers prepared for and conducted telemedicine encounters was more limited and required a semi-structured interview data collection approach. Information collected from both provider groups focused on the advantages and disadvantages of telemedicine versus face-to-face encounters and the tools and resources that can make the conduct of telemedicine encounters easier and/or more effective.

### Data analyses

Participant interviews were recorded and transcribed. Interview transcripts were independently coded by the research team in teams of two (CC, DH, JF, SJ). The coders met to discuss and resolve coding differences through consensus. The study PI (CC) resolved any coding discordance. Transcripts of interviews conducted with the nursing administrative staff participants were analyzed using two different qualitative methods. Coders utilized a structured checklist to enumerate data [12] during analysis to characterize NH challenges with implementing telemedicine during the COVID-19 pandemic and facility plans for continuing telemedicine services after COVID-19. Directed content analysis [13] was used to characterize the advantages and disadvantages of telemedicine when delivering primary care or sub-specialty care. A matrix display [14] technique driven by the Systems Engineering Initiative for Patient Safety (SEIPS) model [15] was utilized to identify and characterize the barriers and challenges to the conduct of telemedicine encounters in participating NHs. Directed content analysis based on the APP structured survey items was used to code the sub-specialist provider and NH interview transcripts followed by inductive coding for additional themes. Following an independent analysis of each data source, qualitative and quantitative results were integrated [16] to characterize the level of agreement between the three groups of subjects with regards to the advantages and disadvantages of telemedicine and the types of tools and resources that can make the conduct of NH telemedicine encounters easier and/or more effective.

## Results

A total of 27 individuals participated in this study, including 12 NH staff, 8 long-term care APPs and 7 sub-specialty care providers (Psychiatry [*n* = 3], Infectious Diseases [*n* = 3] and Wound Care [*n* = 1]). There was limited utilization of telemedicine in two of the participating NHs prior to COVID-19. In both cases, telemedicine was used in an ad hoc manner by mental health providers to address acute resident behavioral disorders. The other participating NHs had not employed telemedicine prior to COVID-19. All participating NHs expanded or implemented telemedicine services during COVID-19. All participating NHs reported encountering difficulties with one or more aspects of telemedicine expansion or implementation. Eight of 9 (78%) NHs experienced issues related to hardware and equipment availability/supply. Connectivity problems as well as software issues were experienced by 89% of NHs. Finally, all NHs experienced challenges with sufficient staff availability and different procedural tasks related to telemedicine (e.g., training staff on use of different telemedicine platforms). Many of the technological issues improved during the early implementation of telemedicine in NHs. However, many of the work system issues, as will be shown, remained a persistent problem in participating NHs. Despite these ongoing challenges, NH administrative staff endorsed positive feelings about continuing telemedicine services after COVID-19 albeit in a more limited fashion.

Interviews with NH administrative staff revealed mixed perceptions about the value of telemedicine for primary care provider (PCP) encounters (Table 1). Participants felt that routine PCP encounters should not be conducted by telemedicine when they could otherwise be conducted on-site. However, if the choice was between an off-site versus telemedicine encounter, participants expressed a preference for telemedicine given resident and staff burdens associated with off-site transfers, particularly among residents with cognitive impairment. NH staff felt telemedicine improved the timeliness and effectiveness of PCP assessment of residents experiencing an acute change-in-condition although one participant felt there was a tendency for some providers to over-utilize telemedicine for issues previously addressed easily by telephone. Finally, several participants noted having PCPs in the building enhanced provider situational awareness and created opportunities for staff education that were lost during the peak of COVID-19.

**Table 1:** Nursing home leadership staff perceptions about the value of telemedicine

**Primary Care Encounters**	**Representative Quote** (A = agreement) (D = disagreement)
**Telemedicine is not a good substitute for routine on-site encounters** *Of the 9 facility interviews, agreement was identified in 6 transcripts, agreement and disagreement was identified in one transcript, and the remaining two transcripts were silent*	(A): We certainly don’t want them to be a replacement for the physician being in the building. (Facility A) (A): It’s important for them to see, have a face-to-face, onsite assessment with that physician, … because we do have a lot of complex patients, and … the doctor really needs to put their eyes on them. (Facility E) (D): Perhaps just those routine visits where they’re reviewing their blood sugars, … [and] blood pressures, they’re doing those things that wouldn’t otherwise require a physician visit. It’s probably just as helpful. (Facility A)
**Telemedicine can enhance the efficiency and effectiveness of acute resident change-in-condition assessments** *Of the 9 facility interviews, agreement was identified in 5 transcripts, disagreement was identified in one transcript, and the remaining three transcripts were silent*	(A):.If there’s anything that’s urgent, like a cellulitis … we want to quick get in a Zoom visit for, not necessarily have to send someone out, let’s treat them here. Those are very effective for telehealth as well, … and negate transfer to the hospital or an ER visit. (Facility B) (D): Prior to the telehealth, they would call and say … do this … but now with telehealth, [it is] like a special visit that we had to do so that they [provider] could see it. … I don't know that that is a fact …, but I think that they tried to do more telehealth visits than were necessary. (Facility C)
**Using telemedicine to conduct a routine encounter is preferable to off-site face-to-face encounters** *Of the 9 facility interviews, agreement was identified in 5 transcripts and the remaining 4 transcripts were silent*	(A): It’s a burden on the resident to have to leave the facility to go to a doctor’s appointment, … For our residents, they have to be picked up at a certain time. The vans are on a schedule as well. … And then it’s … making sure that they get into that appointment safely. So [telehealth] removed that out of the picture, and they can just be seen in their room, so certainly much easier (Facility H) (A): [Provider] will not come in the building either, so then we have to send people out. And those are the cases where this telehealth has been amazing not to have to send them out in the community right now for their compliance visits. (Facility H) (A): … especially the dementia residents that we have where it’s hard to get them out to the clinics. It’s better for them just to stay in the … atmosphere that they know… (Facility G)
**Having PCPs on-site provides benefits that extend beyond the individual clinical encounter** *Of the 9 facility interviews, agreement was identified in three transcripts and the remaining 6 transcripts were silent*	(S): Other things happen … when physicians come, aside from just seeing the resident. There’s a lot of … staff education that happens when physicians are here. We’re asking them questions. They’re educating us about why things are happening. (Facility A) (S): I think it was very limiting in terms of doctors aren't on the unit. They're not sensing what's going on. (Facility C)
**Sub-Specialty Encounters**	**Representative Quote** (A = agreement) (D = disagreement)
**Telemedicine can enhance resident access to sub-specialty care** *Of the 9 facility interviews, agreement was identified in 5 transcripts and 4 transcripts were silent*	(A): … if you call and say this person needs to be seen because they had this skin issue, getting in sometimes can be six weeks, … where a telehealth visit can be a quick five minutes, and they can see what’s going on … and …we’re on to the path of recovery much sooner that we would have been. (Facility D) (A): Especially as in, I mean, rural areas, it’s getting harder and harder to find physicians that come out. (Facility A)
**Intensity of the physical exam is a determinative factor in whether telemedicine can be substituted for a face-to-face encounter** *Of the 9 facility interviews, agreement was identified in four transcripts and the remaining 5 transcripts were silent*	(A): But if you need that pulmonologist to listen to your lungs, that’s what you miss out on. (Facility B) (A): It changed what they were actually doing during the, our visits. I'm sure they were listening to heartbeat and respirations and bowel sounds and all of that, and that wasn't occurring. (Facility C) (A): I think we’ve seen a lot of infectious disease visits be telehealth and then be easier to obtain. You know, sometimes ID is hard to get into, and having that telehealth option, biweekly or whatnot, reviewing those labs, that kind of thing, is very positive. (Facility B)
**Telemedicine can enhance information exchange and collaboration between the sub-specialist providers and other care team members** *Of the 9 facility interviews, agreement was identified in three transcripts, disagreement was identified in one transcript and the remaining 5 transcripts were silent*	(A): Some of our skilled patients have more than one doctor … following them. So … you’re not having to go in and out … you’re able to just put them all together and, whether it be orthopedic and a heart doctor … because several of our patients are more complex, so you deal with several comorbidities at the same time. (Facility E) (A): I have the PT [and] …. the nurse there. I got to see the person, got to get input from both of them. And had she come in my office, she couldn’t have told me any of that information, so it was actually a better visit for me than it would’ve been in the clinic (Facility D) (A): And so I think it helps [providers] … have better communication actually with the nurses than it is having them out and then seeing if they come back with paperwork. … And we’re also able to give … our little speech of what’s going on, little summary of how they’re doing, … (Facility E) (D): … [the] whole connection is also lost when you need to do … a palliative care consult and … all of these other end-of-life decisions, and the providers only saw … them via telehealth. To me, that's a huge issue, so, and we're missing a whole group of the treatment plan. (Facility C)
**Telemedicine can reduce interruptions in needed rehabilitative care when scheduled appropriately** *Of the 9 facility interviews, agreement was identified in three transcripts and the remaining 6 transcripts were silent*	(A): … our focus is rehab, getting better. If you have to go out to the doctor for a [visit]… it kind of shoots your whole day for therapy services. (Facility B) (A): A lot of times there was physical therapy going on, and we were sort of going in the middle of it. And … we sort of trumped them so then they'd have to just sit down and wait for us to get finished. Many times that was nice, but it… disrupted their schedule and … their ability to do what they had to do as well. (Facility C)

NH administrative staff perceptions about the value of telemedicine for the conduct of sub-specialty encounters were positive (Table 1). Participants felt that telemedicine significantly expanded resident access to needed sub-specialty care services, particularly among facilities located in rural locations. Delivery of sub-specialty services via telemedicine reduced interruptions in needed resident therapies when scheduled appropriately and facilitated a greater level of inter-disciplinary engagement than was achievable with off-site sub-specialist encounters. Nevertheless, participants noted the telemedicine modality was less desirable for conduct of sub-specialty encounters where the physical exam played a dominant role in decision-making.

**Fig. 1:**
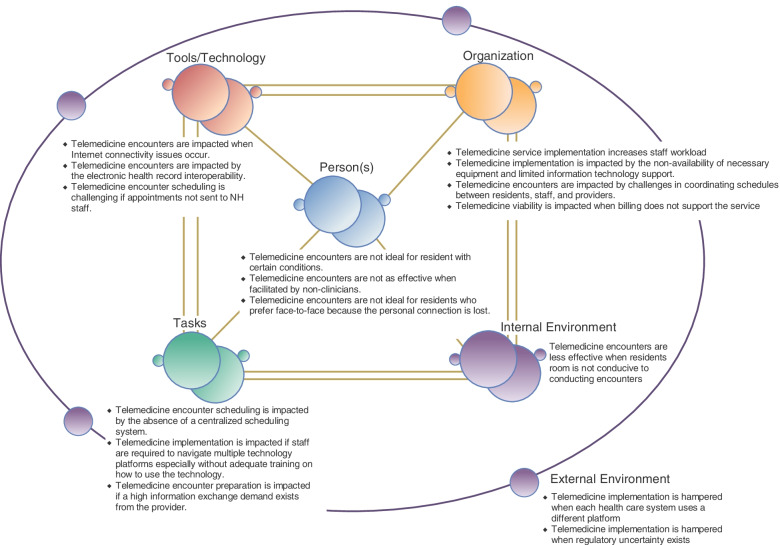
Contextualizing telemedicine work system challenges within the SEIPs 2.0 model

NH administrative staff participants identified a high number of work system factors impacting or impacted by telemedicine (Table 2; see Appendix 1 for supportive quotes). Figure 1 organizes the common barriers or challenges encountered in the context of the work system component of the SEIPs model. Initially, most NHs lacked access to computers and tablets needed for the conduct of telemedicine encounters and several facilities encountered issues with internet connectivity. While these issues improved over time in most facilities, limited inter-operability between NH and provider health system electronic health records (EHRs) remained an ongoing barrier to efficient inter-professional information exchange in these facilities. Participants noted other facets of information exchange extending beyond EHR inter-operability, including unnecessary redundancies as well as significant variation in types of information providers expected and how it was shared, were commonly encountered. Scheduling of telemedicine encounters was a third challenge identified by most participants. Specifically, coordinating telemedicine appointments often required multiple attempts to connect provider clinic and NH staff involved in scheduling decisions. In addition, resident, NH, and staff provider schedules were poorly aligned resulting in limited windows during which telemedicine encounters could be completed and most NHs did not employ a centralized scheduling platform viewable by all staff often resulting in double-booking resident appointments. Finally, introduction of telemedicine created new tasks for NH staff (e.g., pre-encounter information exchange, facilitating encounters, conducting the physical exam). The addition of these new tasks was often not offset with a commensurate reduction in other responsibilities resulting in an overall increase in staff workload. Some NHs were able to accommodate the added workload for short periods of time but often had to rely on complicated cross-coverage schedules or resort to using non-clinical staff for the conduct of telemedicine encounters. This resulted in situations where the person facilitating the encounter was not familiar with the resident’s medical history and/or was unable to effectively perform needed parts of the physical exam.

**Table 2:** Telemedicine work system challenges identified in nursing homes

**SEIPS Category**	**Challenge**	**Further Explanation**
**Tools**	1. Telemedicine platform used by consulting health system sends encounter invite to resident rather than NH staff	• NHs couldn't access resident's email or electronic patient portal to obtain visit links for telemedicine encounter • Many consulting providers lacked access to NH EHR
	2. Internet connectivity issues	
	3. Lack of EHR interoperability between NH & health system	
**Tasks**	1. Difficulty scheduling telemedicine encounters	• NHs often lacked access to a centralized scheduling system/process • Providers and/or their clinic staff had to make multiple attempts to contact resident’s nurse in order to schedule a telemedicine appointment • Multiple NH staff had the ability to schedule resident appointments resulting in residents being double-booked (e.g., physical therapy session and telemedicine encounter)
	2. Training staff on new technology	
	3. NH staff had to learn how to navigate different telemedicine platforms	
	4. High information exchange demand from provider	• Many providers, even those with NH EHR access, preferred to receive information verbally from the NH staff • NH staff often required staff to provide the same information to the provider’s clinic staff prepping the encounter and again to the provider at the beginning of the encounter
**People**	1. Telemedicine encounters are less effective for residents with auditory, visual, and/or cognitive impairments	
	2. Telemedicine encounters were less effective when facilitated by a non- clinical staff member	• Limited availability of clinical staff prompted facilities to use non-clinical staff to facilitate telemedicine encounters • Non-clinical staff unable to provide same level of information exchange as clinical staff and were unable to perform critical aspects of the physical exam
	3. Telemedicine results in a loss of personal connection	
	4. Some residents prefer face-to-face visits	
**Organization**	1.Telemedicine services increased NH staff workload	• Telemedicine created new tasks (e.g., prepping, facilitating) that were simply added on top of other resident care responsibilities
	2. Access to appropriate types and/or amounts of equipment to conduct telemedicine encounters effectively	• Facilities often lacked access to the most effective equipment for conducting telemedicine encounters • Facilities lacked the financial resources to purchase needed equipment • Equipment used for other purposes was often repurposed for telemedicine encounters
	3. Challenges with coordinating resident, staff, and provider schedules	• Telemedicine encounters benefited most from having a clinical staff member present, but these individuals often had competing responsibilities • Provider clinics often requested encounter times that conflicted with critical facility meetings (e.g., morning standup) and resident care activities (e.g., physical therapy)
	4. Limited IT support	• Nursing home staff were often hindered by a lack of support from internal or corporate information technology staff especially as it related to the limited access to the telemedicine software
	5. Billing Issues	• NHs could only submit reimbursement for successfully implemented telemedicine visits that were conducted by video. Encounters where providers directly called the resident or who converted from a video to telephone modality precluded submission of an origination charge • Perception that some providers used telemedicine as a billing opportunity
**Internal Environment**	1. Resident rooms were not ideal for conducting telemedicine encounters	• NH staff felt that the physical aspects of the resident’s room including the absence of furniture to support equipment positioning to allow the provider to see the patient along with poor lighting and the small screen size combined to impact the quality of the telemedicine encounter
**External Environment**	1. Each Healthcare system utilized a different platform	• Since each health care system utilized a different telehealth platform, the impact on NH staff was significant because they had to learn different scheduling systems, different telemedicine platforms, and to understand the preferred clinic telemedicine visit preparation requirements
	2. Uncertain regulatory environment	• NHs were initially uncertain about HIPAA and privacy requirements surrounding telemedicine and whether their facilities were covered

*NH* Nursing Home, *EHR* Electronic Health Record

Data collected from surveys of APPs and interviews with sub-specialty providers revealed areas of agreement and disagreement between groups with regards to the advantages and disadvantages of telemedicine (Table 3). Both groups acknowledged that telemedicine assessments were inferior to those performed face-to-face and they also identified scheduling of encounters as difficult. Participants from both groups also noted that conducting telemedicine encounters increased NH staff workload, that staff facilitating encounters were often unfamiliar with key aspects of the patient history and that lack of access to this information degraded the quality of the encounter. The APP participants identified maintaining continuity of care under quarantine conditions and reducing resident and provider travel as significant advantages of telemedicine. Despite these advantages, APPs were more likely than sub-specialist to express a preference for face-to-face encounters although APP participants noted that telemedicine was a beneficial modality for addressing certain routine resident issues (e.g., discharge planning). APPs were also more likely than sub-specialists to identify deficiencies with the person facilitating the telemedicine encounter, including limitations in their capacity to perform key aspects of the physical exam and their overall familiarity with the resident.

**Table 3:**
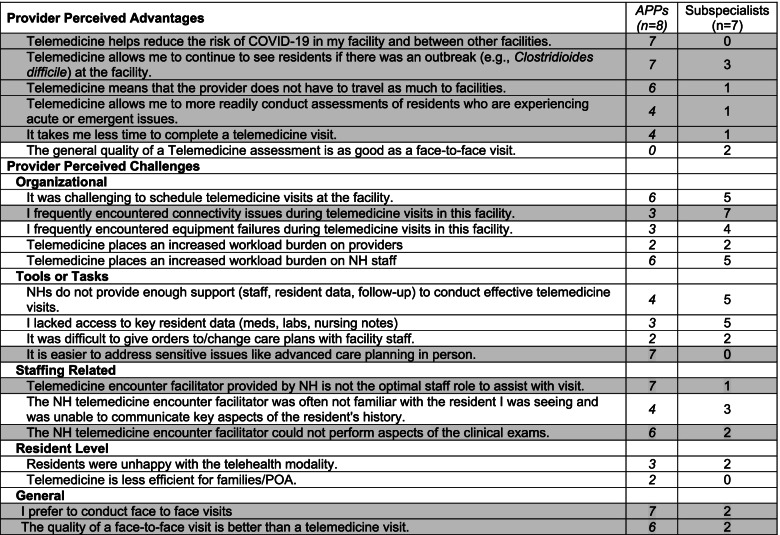
Provider perceived advantages and disadvantages of telemedicine in nursing homes

NH Nursing Home, POA Power of Attorney

Shaded rows represent differences in concordance between APPs and subspecialist regarding the specific advantage or disadvantage of telemedicine use in the Nursing Home

NH administrative staff, APPs, and subspecialists perspectives on the enhancements needed to improve NH telemedicine programs are outlined in Table 4. Participants identified a need to enhance telemedicine equipment/infrastructure, scheduling, information exchange, and the telemedicine encounter facilitator role. External speakers to enhance the encounter audio volume/quality and a telehealth-ready stethoscope were two of the equipment enhancements mentioned by study participants. Nursing home administrative staff and APP participants identified a need to improve telemedicine scheduling systems/procedures and centralize responsibility for scheduling-related tasks. Giving providers access to the NH EHR and standardized protocols for the conduct of telemedicine encounters were two enhancements for improving the quality of inter-professional information exchange identified by study participants. Finally, there was considerable consensus among participants that responsibility for facilitating telemedicine encounters should be centralized to a limited number of NH staff, that clinical staff should facilitate these encounters and non-clinical staff should only be used in secondary supportive roles (e.g., positioning equipment).

**Table 4:** Investments and enhancements needed to make nursing home telemedicine encounters easier and more effective

Category of Enhancements	NH Staff (*n* = 9)	UW APPs (*n* = 8)	Subspecialist (*n* = 7)
**Equipment and Infrastructure**			
NHs should invest in dedicated and adequate/appropriate equipment to conduct telemedicine visit (e.g., laptop or tablet)	6/9	3/8	7/7
Telemedicine visits could be enhanced through purchasing and making available sound amplification devices for use with hard of hearing individuals	5/9	7/8	6/7
Telemedicine visits could be enhanced through the availability of a stethoscope device to conduct remote heart and lung exams	0/9	7/8	1/7
Telemedicine equipment should have enhanced video capabilities to allow for a better view of the patient on camera	2/9	N/A	2/7
NHs should invest in the infrastructure necessary to support telemedicine visits through improved connectivity and bandwidth	1/9	5/8	7/7
**Scheduling**			
NHs should develop or invest in an improved and standardized system for scheduling telemedicine visits	7/9	5/8	2/7
NHs should designate a point person to schedule telemedicine visits	6/9	N/A	1/7
NHs should consider scheduling telemedicine visits in pre-determined blocks of time with adequate pre and post visit time to allow for NH staff prep and follow-up	3/9	N/A	2/7
**Information Exchange**			
NHs and providers should work together to provide remote access to NH electronic health records to facilitate telemedicine visit preparation and pre-charting activities	4/9	4/8	2/7
NHs should create policies and procedures that template the expectations about how a telemedicine should be conducted	2/9	N/A	3/7
**Telemedicine Encounter Facilitator**			
NHs should identify and dedicate staff to facilitate telemedicine visits	6/9	7/8	6/7
NH telemedicine encounter facilitator should be a clinician (I.e., RN or LPN) who can conduct telemedicine visit requested assessments making the visits more efficient and effective	5/9	7/8	6/7

*APPs* Advanced Practice Providers, *NH* Nursing Home, *N/A* Not applicable because the code or item was not addressed in the quantitative APP survey

## Discussion

The COVID-19 pandemic resulted in a rapid expansion of telemedicine in NHs. The NHs in this study encountered numerous challenges with implementing telemedicine services at the beginning of the pandemic. While improvements in some areas were observed, most of the participating NHs reported experiencing ongoing work system challenges that degraded the quality and effectiveness of telemedicine encounters. These work system challenges aligned with findings from other studies that identified efficiency and workflow, staff training, interoperability, and cost as barriers to global telemedicine adoption [17–19]. Despite these issues, participating NH staff and providers held positive perspectives on the value of telemedicine. Moreover, most participants were supportive of continuing telemedicine use in NHs, particularly if coupled with enhancements to the tools and systems used to deliver and support these encounters.

The current study adds to the existing literature examining NH staff and provider perceptions about the value and benefits of telemedicine [10, 20–22]. Avoidance of resident transfers to off-site clinic locations was one of the biggest benefits of telemedicine identified and is consistent with other published studies of telemedicine in NHs [21–23]. Providing NH residents with timely access to sub-specialty services was the other major benefit of telemedicine identified in the current study although participants felt this was highly contingent on the importance of the physical exam in the assessment process. Our findings are somewhat at odds with a previously published study which identified several highly physical exam dependent sub-specialties (e.g., cardiology and neurology) as among the most highly valued services [21]. None of the NHs that participated in the current study had access to a telehealth-ready stethoscope which may partially explain these differences despite evidence that use of such medical devices may improve provider decision making [24]. A third major finding of the current study is that participants expressed a clear preference to conduct routine primary care services on-site rather than by telemedicine. While other comparisons of the parity of telemedicine with face-to-face encounters in NHs are limited, one study focused on wound care delivery in NHs identified a similar preference for on-site care delivery [25].

The current study adds to growing literature on implementation of NHs telemedicine programs during the COVID-19 pandemic [26, 27]. Challenges with internet connectivity, limited access to dedicated equipment, staff familiarity and comfort with using different telemedicine platforms and availability of staffing to conduct tasks related to the conduct of telemedicine encounters were identified in the current study. Similar to other studies examining telemedicine implementation in NHs prior to COVID-19 [22, 28–30], participating NH administrative staff noted that many of these challenges, particularly those related to technologies and their use, improved greatly over time. Nevertheless, challenges related to scheduling telemedicine encounters, inter-professional exchange before, during and after telemedicine encounters, and balancing clinical staff workload around telemedicine and other resident care tasks remain persistent problems a year and half into the COVID-19 pandemic.

As has been argued by others [31], NH telemedicine regulations that were relaxed during COVID-19 is a necessary requirement for sustaining widespread telemedicine utilization in NHs after the pandemic. Moreover, it is reasonable to expect that the technology for conducting telemedicine encounters as well as the costs to acquire these tools will continue to improve which will further enhance adoption and sustainment of telemedicine in NHs. However, the challenges of integrating telemedicine into existing NH work systems and its ongoing effects on facility and off-site clinic workflows are barriers that cannot be easily alleviated by policy or purely technological solutions.

Enhanced collaborative relationships between NHs and the off-site clinics that provide telemedicine services to the NH is a critical need. Actions that can be taken now, include: 1) providing NH staff and off-site clinicians with ready access to each other’s EHRs; 2) requiring that different health systems providing telemedicine services to the same NH agree to utilize a common telemedicine platform to reduce the number of platforms that NH staff must be familiar with; 3) development of protocols that standardize the content, structure and sharing of information between NH staff and providers involved in telemedicine encounters; 4) centralizing scheduling related tasks in the NHs and ensuring that these individuals are easily reachable by staff in off-site provider clinics; and 5) centralizing responsibility for facilitating telemedicine encounters to clinical NH staff who are given sufficient time to collect and prepare information needed before, during and after the conduct of an encounter. Further improvements will likely be realized through policies to promote greater inter-operability between NH and health system EHRs, greater adoption of technologies that allow cross-organizational scheduling and promote real-time communication between NH and off-site clinic staff responsible for resident scheduling decisions, and the introduction of novel funding mechanisms that provide NHs with the resources needed to adopt and sustain telemedicine technologies as well as hire and retain the staff needed to ensure its reliable delivery.

Health care systems including providers, hospitals, and nursing homes could address the identified challenges by implementing enhancements needed to make nursing home telemedicine encounters easier and more effective [32]. Some recommendations such as interoperability, appropriate equipment, and education and training align with existing recommendations related to telemedicine use in nursing homes [33, 34]. Successful implementation would allow nursing homes to address domains in the National Quality Forum measurement framework for telemedicine such as access to care for the patient, family and care team and the effectiveness and experience of the telemedicine encounter [34]. Although not a focus of this study, evidence suggests that the use of telemedicine in nursing homes improves resident health, and reduces adverse drug events, hospitalizations and emergency room transfers [7, 28, 31, 35–38].

The project had several limitations. The interviews were conducted with a convenience sample of NH staff and providers and the viewpoints expressed may not be representative the experience of other NHs that adopted telemedicine during COVID-19 or the plans of other facilities for continuing its use after the pandemic. A statewide or national survey would provide insights from a more representative sample about prior experiences and future plans about telehealth utilization in nursing homes. Sub-specialist interviews were limited to a convenience sample of infectious disease, psychiatry, and wound care providers. The perceptions about telemedicine benefits and challenges are not generalizable to other nursing homes due to the study sample size especially for specific sub-specialists (e.g., wound care). Other sub-specialists may have different perspectives about the value of telemedicine and the enhancements needed to improve its delivery and effects. Future research should focus on conducting a wider sample of sub-specialist interviews including multiple perspectives within the same sub-speciality. Finally, the findings from the survey were limited to one long-term care APP group affiliated with a mid-western healthcare system. The survey should be replicated across a larger sample to determine the representativeness of the results observed in the current study.

In conclusion, nursing staff and providers who deliver care in these facilities recognize that telemedicine is a valuable service modality that they feel should be continued. Many of the facilities participating in this study were able to enact changes to structure and process that enhanced their telemedicine workflows over time. However, facilities still face several ongoing internal and external environmental challenges that threaten the sustainability of telemedicine after COVID-19 recedes. Regulatory relief, new payment models and incentives to encourage greater collaboration between NHs and the healthcare systems that participate in facility telemedicine services will be needed to avoid a return to the pre-COVID-19 status quo.

## Supplementary Information

**Additional file 1.**

## Data Availability

The datasets used and/or analyzed during the current study are available from the corresponding author on reasonable request.

## Abbreviations

APP: Advanced practice providers
CMS: Center for medicare and medicaid services
COVID-19: Coronavirus disease 2019
EHR: Electronic health records
ER: Emergency room
ID: Infectious disease
IT: Information technology
NH: Nursing homes
PCP: Primary care provider
PI: Principal investigator
POA: Power of attorney
RN: Registered nurse
SARS-CoV-2: Severe acute respiratory syndrome coronavirus 2
SEIPS: Systems engineering initiative for patient safety
U.S.: United States
