# Supplementary material for: There and back again: the shape of telemedicine in U.S. nursing homes following COVID-19
Source: BMC Geriatr. 2022 Apr 19;22:337. doi: 10.1186/s12877-022-03046-y (PMC9015887; doi:10.1186/s12877-022-03046-y)
Supplement: Supplementary file 1 — Additional file 1. [file 12877_2022_3046_MOESM1_ESM.docx]

**Appendix (Supplementary Materials)**

**Table 2: Telemedicine work system challenges identified in nursing homes (with supportive quotes)**

| **SEIPS Category** | **Challenge** | **Further Explanation** | **Quotes** |
| --- | --- | --- | --- |
| **Tools**  **Tools** | 1. Telemedicine platform used by consulting health system sends encounter invite to resident rather than facility staff. | - NHs couldn't access resident's email or electronic patient portal to obtain visit links for telemedicine encounter. - Many consulting providers lacked access to NH EHR. | “Like (Health System 1) and (Health System 2) will only allow a televisit via MyChart. And many of our long-term care residents don’t have MyChart, and their children, who sometimes are in their 70s, are not, you know, really hip to MyChart either. So that becomes an issue.” -Facility E  "And also there was clinics where they only wanted to go through their MyChart, the patient’s MyChart. We couldn’t, we can’t access that here, especially with our demographic on top of it all. We would have to, if the clinic could not send a link to the nurse manager, we were to a point where we couldn’t complete the visit. And in some cases, we couldn’t do telephone either because the cognition of the resident, which it was near impossible to even complete that way. Those were, the MyChart was a challenge. And it’s definitely more now that the clinics more and more, as we’re opening more up, they want it all through MyChart. They do not want to be sending links to a different source."  (Facility H)  “some of the hospitals, actually (Health System 3) for a while would only set up telehealth, and they would send the link to the patient’s email. And we’re like, but they don’t have email, and we’re not setting up email accounts for everybody for this because they can’t access it."  (Facility D) |
|  | 2. Internet connectivity issues |  | “they'd lose connection, and then they'd have to come back on, and it was kind of, or doctors who are working from home until it kind of got going and people got what they needed.  You know, the connections weren't quite as good, especially if they're rural.” (Facility C)  ”The other thing is the Internet service, it tends to freeze, where a doctor, again, just like we had here going this morning, you know, that type of issue, and the clock is ticking. “ (Facility E)  “we had a few glitches, some connectivity problems at first until we figured that out.”  (Facility A) |
|  | 3. Lack of interoperability between NH & health system EHRs |  | “Two different. Unfortunately, two different. So actually getting pictures into our system, our (NH EMR system 1), is much more difficult because of the restrictions of IT. So you have to have specific USB rights to be able to upload pictures. So we would have to upload it into the computer, save it into a file, and then you could upload it into (NH EMR system 1) But you have to have those rights, and they don’t just give out those rights readily. You know, basically I have three people in the building that have that right to be able to do it. I, my nurse managers don’t have the right to be able to do that."  ( Facility H) |
| **Tasks**  **Tasks**    **Tasks**  **Tasks** | 1. Difficulty scheduling telemedicine encounters | - NHs often lacked access to a centralized scheduling system/process. - Providers and/or their clinic staff had to make multiple attempts to contact resident’s nurse in order to schedule a telemedicine appointment. - Multiple NH staff had the ability to schedule resident appointments resulting in residents being double-booked (e.g., physical therapy session and telemedicine encounter). | "I kept a master schedule through a spreadsheet that had everybody’s telehealth that would come through. There was also other unit clerks that would schedule telehealth, and I would enter them in the spread-sheet, so make sure nothing was conflicting. If it was, we had to reschedule. But every week, I would send out an updated list, so then the nurse managers would put it in their personal calendar. So whenever there was a new visit that might pop up on the next day, I would personally email them so they’re aware. It was put in our electronic system, but it wasn’t always, they wouldn’t always be able to catch them the way they’re put in for them necessarily to see. So I always wanted to make sure they were up-to-date on who was being seen. So I kept the master.   (Facility H)  “… the scheduling and just finding how to schedule it so it doesn’t conflict, knowing kind of what these nurse managers and what’s going on in their days as well. Only having, I was pretty much mainly doing all the scheduling, which helped. But then later on when things, as they lift, in the fall, the other unit clerks would help schedule, but they would also have to watch the schedule, what I’m scheduling, to make sure they weren’t conflicting." (Facility H)  "… initially, the televisits were kind of like whenever, kind of just scheduled when the doctor kind of wanted. And then again, we have to have that kind of discussion in saying, this doesn’t really work in our world, because it’s not, you know, again, not like a clinic where we start at this time, or this is all we’re doing all day. Our nurse managers are doing multiple different things. So, and they have certain routine or daily meetings that take place, and we really don’t want to conflict with that."" ""So for example, a daily nursing stand-up meeting, well, can a nurse manager miss it? Sure, they could, but then that’s a piece of information that I’m missing as the DON, so it’s difficult for me to report off on what’s going on and know what’s going on within the facility if they miss that meeting. So I wouldn’t want them missing that routinely. Like I said, obviously it can happen from time to time."  (Facility H)  'And then having the clinic running on time and another wrench in it, not every clinic has scheduled certain ways to make all these visits work conveniently where you could just have one person schedule and do this all the time. One clinic . . . only operate on Tuesdays to do visits. Other days are surgery days. So that, the scheduling from the clinics throws the wrench in it as well, and when the providers are available too." (Facility H) |
|  | 2.Training staff on new technology |  | "Nurses, by virtue, we aren’t all the most electronically, we’re a little electronically challenged, some of us, especially us old ducks. So having a better comprehension, I mean, we actually grabbed the CNAs a few times. We’re like, okay, we need your help. You’re young. You understand this. And they were able to help us through it." (Facility D)  So we all had to quickly learn how to operate iPads …" (Facility D)  "But just learning some of that part, how to handle the equipment to make that, you know, actually work and be more proficient, but I think we’ve got that part down now." (Facility D) |
|  | 3. NH staff had to learn how to navigate different telemedicine platforms |  | The staff seemed to be confused about the different entities using different programs, like (Health System 3) using, and I couldn’t even tell you what they were right now, but each one had a different program they used.  And that’s just information frontline staff don’t retain or then know how to navigate.  So and I’m not sure how it could’ve been more streamlined, but, you know, each clinic seemed to have their own process.”  (Facility B)  "And that was a little bit difficulty with technical support on getting, you know, (Health System Video Platform 1) available for, easily accessible for our staff to be able to use for those visits."  (Facility H)  The apps can be challenging … sends an email link, and then it’s from the email link, and then you have to log onto that, and then you have to share it, and then it has to be opened in Chrome, and then ultimately, you get to the website, which is very cumbersome. And when you have glitches, it just makes it even more so. Sometimes the audio connections can be glitchy". (Facility E) |
|  | 4. High information exchange demand from provider | - Many providers, even those with NH EHR access, preferred to receive information verbally from the NH staff. - NH staff often required staff to provide the same information to the provider’s clinic staff prepping the encounter and again to the provider at the beginning of the encounter. | “I know my nurse managers have expressed that some physicians will not start the meeting until that prescreening has been done. And again, we feel as that this is a different setting. You know, we’re in a professional setting. We have that information. We can give you that information. It’s, you’re not dealing with, you know, somebody off the street who you have to go through all these steps of questions before we can even start this visit. And again, that just slows things down." (Facility H)   “I know the nurse managers felt like the clinician didn’t do their part before the meeting, because it’s like, you had all this information. We provided it to you. If you would’ve read through it, you would have this information, versus now we’re having the meeting, and you’re asking me questions, and you want me to look up this and look up that, which I’ve already provided to you a day or so ago." (Facility H)  "It is my standard to get fax numbers and fax two days prior the list of their medications, current vitals, and any other pertinent information that the nurse manager or that has been requested. And then I put on the fax cover sheet to call the team nurse for any other further information prior to, for intake for the appointment. I felt like sometimes with that, with a certain clinic, it just turned a blind eye. They never looked at it ever. And it just, it really became time consuming for the nurse managers. And these visits may end up being 40 minutes at times because they didn’t take the time to do their review of items that we have already gave to them.""    (Facility H)  “… clinicians sometimes just want you to do it for them versus looking it up themselves. So can you just give me the blood sugars for the past two weeks? Well, it’s, you know, you have access. Can you look that up? You know, unfortunately we don’t have, it’s not like we have a nurse designated for these visits or like a clinic where you have a nurse that’s working with you or a couple of medical assistants that are getting this information, providing it to you or whatnot."" (Facility H)  "To some extent, especially with the televisits, we did start having like a standard, ahead of time, this is the information that we’re going to gather, because, again, we found that these are the types of things that were being asked, and we were hoping to try to streamline the visits, so they didn’t take as long. Some practitioners were better at . . . at the information that’s provided than others. Some, just like [name] said, seemed like they just never received it, and they’re asking you to do everything again." (Facility H) |
| **People**  **People**  **People** | 1.Telemedicine encounters are less effective for residents with auditory, visual, and/or cognitive impairments |  | “… memory care definitely has been the area that tele-health is not ideal because we’re working with a resident that isn’t able to really speak for themselves. So it’s best to really have a personal visit. They’re not as productive. I mean, I think those types of visits went much quicker because there’s not much the physician can do or the GNP can do with the resident with, you know, memory impairment. They’re going to rely upon the data that they have. And doctor, one of the doctor’s that manage, [physician] who did the memory care, had mentioned that too. He said it’s just harder not to be there with his residents and in person. You’re not getting everything you need from these visits."" (Facility H)  “I think overall, as far as the residents’ challenges, it would’ve been hearing or seeing them or maybe just kind of struggling with understanding what was happening at first. (Facility A) .Although, we noticed that with residents with dementia, we had to be careful that they weren’t frightened by voices coming out of this strange-looking contraption. So that was something we had to be mindful of as well.  The disadvantages, it’s hard for some of the residents to see or hear the physician.” (Facility A)  “The dementia I mentioned.  They were very confused, hard of hearing, looking at me versus the provider, yeah, and then just not sometimes giving the residents enough time to process before responding” (Facility C) |
|  | 2. Telemedicine encounters were less effective when facilitated by a non- clinical staff member | - Limited availability of clinical staff prompted facilities to use non-clinical staff to facilitate telemedicine encounters. - Non-clinical staff unable to provide same level of information exchange as clinical staff and were unable to perform critical aspects of the physical exam. | “I'm not clinical in any way, shape, or form, which is sort of a limitation”. (Facility C)  “But if you need that pulmonologist to listen to your lungs, that’s what you miss out on. There are some telehealth units that have the Bluetooth stethoscopes and that kind of thing. We don’t have that.” (Facility B)  “Hospice visits.  I was doing those.  And, you know, it was really hard because I'm, they're asking me questions.  How is their breathing?  Are they doing this?  Is it different than yesterday?  And I don't really know that, and that was very limiting.”  (Facility C)  “Because I wasn't clinical, that was a challenge, and it was, it changed what they were actually doing during the, our visits.  I'm sure they were listening to heartbeat and respirations and bowel sounds and all of that, and that wasn't occurring.” (Facility C)  “Like sometimes they'd be like, well, can you pull their sheets back so I can see their feet?  Are their legs swelling?  And I don't know that information because I'm not clinical.  So that was sometimes very, very limiting.”   (Facility C) |
|  | 3. Telemedicine results in a loss of personal connection |  | “… the assessment and the personal touch is lost. So definitely the assessments, I think any practitioner would prefer to do the assessment their self, them-selves, but obviously that can’t happen." (Facility H)  “I also think that we are forgetting one of the most important aspects of physician visits is families.  So, you know, my mother and my father-in-law both lived in the nursing home and then died in the year of COVID.  And, you know, so for my mom to see the doctor come in and say to me, honey, the doctor came in and saw me, was very important for her, so I think, and then that made our family feel better.” (Facility C) |
|  | 4. Some residents have a preference for face-to  face visits |  | There are some appointments, you know, like with a cardiologist that that resident really wants to have a one-on-one conversation with, I’ll use as an example.  Maybe that is a, needs to be in person. (Facility B)  'The geriatric population likes to see their physicians. And then I think that’s going to be the biggest player.  Our NPs are great.  Even when they come in, you know, the res-ident will say, well, where’s my doctor?  And they have to give the spiel. The doctor doesn’t come here. I, you know, you know that they’re not used to that necessarily.  But I do think that resident choice will drive it." (Facility B) |
| **Organi-zation**  **Organi-zation**  **Organi-zation**  **Organi-zation** | 1.Telemedicine services increased NH staff workload | - Telemedicine created new tasks (e.g., prepping, facilitating) that were simply added on top of other resident care responsibilities. | “Sometimes, unless they were busy and it was kind of a pain for them to have to sit in on the visit.  (Facility G)  "I think one of the biggest challenges is the time, is having somebody available and working that into their day to see that, to work with that resident, work with that physician, and sticking to the time frame. I don’t think they really stuck to the time frame." (Facility H)  "Well, that was tough with the clinics because they’re still running on clinic time, that they might be running 15 minutes late. And typically when you’re at a doctor’s office, you’re, it’s just the way it is. And it would affect here when the staff is ready to go and, unfortunately, the time to wait, there was times where I spent 30 minutes, and they didn’t even connect yet. And then all the sudden, half an hour later after that they, want to connect. And it’s unfortunate, because sometimes, we don’t have the availability. So that could’ve been, that was frustrating."   (Facility H)  “But with the telehealth visits, the nurse has to stay with the resident the whole time usually, because either they can’t hear very well, or the doctor has questions, and so it’s a little bit more time consuming for the nurse.” (Facility G)  "... And obviously, a telehealth visit, if we’re lucky, is only going to be 15 minutes. But quite often, it’s 20 to 30 minutes that it’s taking. And of course, that, depending on the resident, that nurse might need to stay in that room the entire time. Now, if it’s a different type of conversation, and the resident is more with it, of course, the nurse doesn’t have to stay in that room the entire time, but they would have to return, say, in 10, 15 minutes and then answer whatever questions and take whatever orders that the physician may have." (Facility H)  "… they’re [PROVIDERS] really reliant on the nursing staff to be able to provide what are acute issues going on that need to be addressed, and there may be information that is lacking and not getting through with having this process with telehealth versus the provider coming in, being able to make rounds, and interacting with the nursing staff and asking them questions. I think it flows better in person versus the telehealth." (Facility F) |
|  | 2.  Access to appropriate types and/or amounts of equipment to conduct telemedicine encounters effectively. | - Facilities often lacked access to the most effective equipment for conducting telemedicine encounters. - Facilities lacked the financial resources to purchase needed equipment. - Equipment used for other purposes was often repurposed for telemedicine encounters. | “…probably the hardest part was just getting me the right equipment, which was a laptop with audio and a video.”  (Facility C)  “We were able to get a little, some additional equipment, so we would, you know, we were able to more readily, we, at first we didn’t have a lot of that equipment available either, so we did some purchasing along the way as well.” (Facility A)  “I was taking a valuable piece of equipment away from a CNA usually during a time when they actually had time to sit down and enter their feedback.  And so when I'd walk in, they'd have to give it up, and that was really, I hated to see that because I know they don't have a lot of time sometimes to enter that kind of data.” (Facility C)  “And getting the laptop with the right video and audio on it made a difference.” (Facility C)  “My phone was a really old phone, and so for certain things, it didn’t work. So for (Health System 3), they use their own special thing, so then I had to get an iPad.” (Facility G)  "… it’s just equipment in general, having the good equipment. I mean, for example, you know, laptops are out there, but there’s older laptops, and there’s newer laptops. Obviously, the older laptops are clunkier. The speakers aren’t as good. But that might be all that person has in that area. So having, you know, good equipment all the time, but also like a good set up." (Facility H) "We did purchase iPads to assist with telehealth visits. And, of course, we have laptops that we purchased as well." (Facility H)  "As time went on, we, you know, learned that some lap-tops or iPads just aren’t the, aren’t loud enough for the residents to hear." (Facility H)  “So there is definitely a need to have a different speaker system to use. And we’ve been using the Jabra speakers. Several nurse managers have that, and that’s greatly helped the residents hear and understand during the visits." (Facility H)  "when they’re in the rooms with the laptops too, sometimes the residents have a hard time seeing on them. So it’s like when [name] said having that separate room, if you had a bigger screen for the residents to see, I think that would be better for them as well, because their vision isn’t as well. And these little screens sometimes, and I just, I know like, and some of them are trying to read their lips too, some of the doctors, with the ones that don’t hear as well." (Facility H) |
|  | 3. Challenges with coordinating resident, staff and provider schedules | - Telemedicine encounters benefited most from having a clinical staff member present, but these individuals often had competing responsibilities. - Provider clinics often requested encounter times that conflicted with critical facility meetings (e.g., morning standup) and resident care activities (e.g., physical therapy). | I had some people where they'd call the patient directly, the resident directly.  They answered all the questions, and then they're like, oh, no one is here to do the video, so then they just did their call by phone instead of virtually So sort of as some of those happened I got a little bit more like, oh, I've got to get up there like a half an hour before I've got to tell everyone on the unit that I'm here to help with the call because we don't know who is going to answer the phone.” (Facility C)  “Each clinic seemed to have their own process.  You know, and some clinics called before, some didn’t.  Some, if it could’ve been more standardized, this is the process it would’ve been easier for frontline staff.” (Facility B)  “Some of the clinics, like I said, wanted staff available to answer questions beforehand. Some of them wanted that nurse to come with us to the visits, having to be available sort of 20 minutes ahead.” (Facility C)  “But it's, it was very challenging in terms of when it was sort of, like I did one this morning. It was an atypical call. It was a telehealth, but it was with (Health System 4) but not our usual. So sometimes they, (Health System 4), like they'll say appointment is at 10:30. They want to call about 20 minutes prior, so you have to be ready about ten after. And they're going to call, and they're going to speak to somebody, and then at 10:30 the . . . is going to get on the phone. But on our units, sometimes they called the resident directly, or sometimes they called the nurses' station, or sometimes they called, and nobody answered. You know, there's a lot of people to coordinate. So then I'm running down the hall trying to talk to the resident if they got the phone call, and then they're like, oh, nobody was here, so then we canceled the appointment, or the nurses didn't know that I was coming or, you know what I mean? So sometimes that coordination was really crazy.” (Facility C)  “Sometimes we would have to schedule, I know one specific one we had to schedule something with the wound nurse, myself, the provider, because, you know, they needed to see something.” (Facility C)  “A lot of times there was physical therapy going on, and we were sort of going in the middle of it.  And I hate to say it, but we sort of trumped them so then they'd have to just sit down and wait for us to get finished.  Many times that was nice, but it, I think it disrupted their schedule and, you know, their ability to do what they had to do as well. I think it was very limiting in terms of doctors aren't on the unit They're not sensing what's going on.” (Facility C) |
|  | 4. Limited IT support | - Nursing home staff were often hindered by a lack of support from internal or corporate information technology staff especially as it related to the limited access to the telemedicine software. | "Our MIS department or computer department is kind of like trying to work with the gestapo sometimes. They were very hesitant wanting to give us access to too much stuff, so every time we needed something, they were like, well we have to put it on the iPad. And finally I’m like, you got to find another way to do this. There’s got to be a way you can electronically push these things out. And we need them on all the iPads, not just one." (Facility D)  "And that was a little bit difficulty with technical support on getting, you know, (Health System Video Platform 1) available for, easily accessible for our staff to be able to use for those visits." (Facility H) |
|  | 5. Billing Issues | - NHs could only submit reimbursement for successfully implemented telemedicine visits that were conducted by video. Encounters where providers directly called the resident or who converted from a video to telephone modality precluded submission of an origination charge. - Perception that some providers were using telemedicine as a billing opportunity. | I do know in terms of the billing like I was doing a sheet every week.  And then I would mark off who we actually saw, and I was giving that to our medical biller.  And she only wanted people that there was a physical video connection on.  So like the times when there was a phone call to the resident and we never got the video up, we never, we couldn't bill for that, because that wasn't, or they couldn't bill for it because it wasn't actually a visual, whatever the rules were around that.  If it wasn't visual, she wanted me to cross it off my list.”  (Facility C)  “The provider was having poor connection and it ended up only being a phone call, did that count as a visit and, you know what I mean?”   (Facility C)  “I think some of the clinics did a lot more telehealth than they would have done if they were in seeing the people themselves.” (Facility C)  “I think that they tried to do more telehealth visits than were necessary.  And in the past, they would have never, you know, said, oh, I'm going to come in and see that red toe.”   (Facility C)   “I think they used this telehealth as a billing opportunity and a revenue generation.  And good or bad, right or wrong, that's just, as I said, they would have never done that prior.  Oh, let me schedule a telehealth.  Okay.”  Sure.”   (Facility C) |
| **Internal Environment** | 1. Resident rooms were not ideal for conducting telemedicine encounters | - NH staff felt that the physical aspects of the resident’s room including the absence of furniture to support equipment positioning to allow the provider to see the patient along with poor lighting and the small screen size combined to impact the quality of the telemedicine encounter. | “… even better than that would be an actual room, I think, that’s set up or rooms that’s set up where you could go in, and all that equipment is there, better equipment. As you may know, that, a computer camera, an, you know, a separate computer camera that you plug into your computer is much better than the camera that you get with the laptop. So I would think that you’d want to have something minimum like that that would be better. And of course, the audio equipment is better than a laptop as well. But then, you know, good set up, good lighting, that type of thing. I mean, our rooms, resident rooms, I wouldn’t say that that’s the most ideal lighting. I would say in a clinic, where there, you know, the clinic rooms, the lighting is much brighter. You know, so just having that available would make it a better visit." (Facility H) |
| **External Environment**  **External Environment** | 1. Each Healthcare system utilized a different platform | - Since each health care system utilized a different telehealth platform, the impact on NH staff was significant because they had to learn different scheduling systems, different telemedicine platforms, and to understand the preferred clinic telemedicine visit preparation requirements. | “It was a roller coaster in the beginning, specifically with, well, between being part of a system, (Health System 2), and working with other systems, you know, different systems decided on different avenues to do telehealth. So that was a little bit of a barrier that we had to kind of work through. You know, (Health System 3) wanted to use, (Health System Video Platform 1) I believe it was called, and (Health System 2) wanted to use the (Health System Video Platform 2) okay."  (Facility H)  "… get the different platforms downloaded because, that’s probably been the biggest challenge, is, you know, (Health System 3) has their own platform. (Health System 2) has their own platform. Other doctors’ offices have their own platforms. Some docs were fine with doing, you know, (Health System Video Platform 3) in a cell phone. Some wanted (Health System Video Platform 2)."  (Facility D)  "So in my opinion, the biggest challenge was the electronic side, getting all of that figured out first and getting all of the different platforms available to all the players that needed to have it, in the midst of a pandemic." (Facility D)  "that there is no standard across the systems, that each of the different, you know, groups has their own way.” (Facility E)  "because it’s different not only across each of the three systems but, you know, from physician to physician.” (Facility E) |
|  | 2. Uncertain regulatory environment | - NHs were initially uncertain about HIPAA and privacy requirements surrounding telemedicine and whether their facilities were covered. | "Having some better definitions from a HIPAA perspective, which I know, you know, at some point, the federal government sent out that we were all HIPAA protected because we just had to do what we had to do right now, so that was helpful." (Facility D) |

NH= Nursing Homes, EHR= Electronic Health Record
